# Tumour Necrosis Factor Alpha (TNF-α) and Oral Squamous Cell Carcinoma

**DOI:** 10.3390/cancers15061841

**Published:** 2023-03-19

**Authors:** Gary Brierly, Antonio Celentano, Omar Breik, Elham Moslemivayeghan, Romeo Patini, Michael McCullough, Tami Yap

**Affiliations:** 1 Maxillofacial/Head and Neck Surgery, Royal Brisbane and Women’s Hospital, Queensland Health, Brisbane, QLD 4072, Australia; 2 Faculty of Medicine, University of Queensland, Brisbane, QLD 4072, Australia; 3 Melbourne Dental School, Faculty of Medicine, Dentistry and Health Science, University of Melbourne, Carlton, VIC 3053, Australia; 4 Department of Head, Neck and Sense Organs, Università Cattolica del Sacro Cuore, 00168 Rome, Italy; 5 Fondazione Policlinico Universitario A. Gemelli IRCCS, 00168 Rome, Italy; 6 Dermatology, Royal Melbourne Hospital, Melbourne Health, Parkville, VIC 3050, Australia

**Keywords:** TNF, tumour necrosis factor, cytokine, oral cancer, OSCC, oral squamous cell carcinoma, head and neck SCC, inflammation, OPMD, leukoplakia

## Abstract

**Simple Summary:**

TNF-α is of interest in oral squamous cell carcinoma (OSCC), with its demonstrated presence affecting both tumour and stromal inflammatory cells to enhance proliferation and facilitate invasion. TNF-α gene polymorphisms have also been associated with an increased risk for both oral pre-cancer and cancer development. Here we present a review of the current knowledge of the role of TNF-α in the aetiology, pathogenesis, and potential therapy of OSCC.

**Abstract:**

Uncovering the inflammatory mechanisms underpinning initiation, progression, and promotion of oral squamous cell carcinoma (OSCC) development is fundamental to the rational pursuit of targeted therapeutics. Here we present a review of the current knowledge of the role of TNF-α in the aetiology, pathogenesis, and potential therapies with regards to OSCC. TNF-α is worthy of particular attention in OSCC, with its presence demonstrated to enhance cell proliferation and its downregulation demonstrated to inhibit proliferation and migration in other carcinomas in both in vitro and in vivo models and oral cancer patients. Increased TNF-α in the OSCC tumour microenvironment has been demonstrated to favour invasion through promotion of firstly the pro-inflammatory, pro-invasive phenotypes of OSCC cells and secondly its paracrine mechanism mediating recruitment and activation of inflammatory cells. Polymorphisms affecting the gene expression of TNF-α have been strongly associated with an increased risk for oral squamous cell carcinoma. A number of studies have considered TNF-α within biofluids, including saliva and serum, as a potential biomarker for the early detection of OSCC, as well as its staging, differentiation, and prognosis. The broad and multifaceted role that TNF-α plays in many inflammatory states presents an obvious confounder, particularly with demonstrated increased TNF-α levels in common oral disease states. Lastly, biologic agents targeting TNF-α are currently in clinical use for immune-mediated inflammatory rheumatological and gastrointestinal diseases. There is the potential that these biological agents might have an adjunctive role in OSCC prevention and treatment.

## 1. Introduction: 

Oral squamous cell carcinomas (OSCC) are the most common mucosal cancers of the head and neck [1]. OSCC cases are predicted to rise to 856,000 cases by 2035 in line with predicted global demographics [2]. Globally, the overall five-year survival of OSCC has not improved significantly beyond 50% and less than 1 in 5 patients who present with metastatic disease at the time of diagnosis will survive for 5 years [3]. Surgery and adjunctive radiotherapy remain primary treatment approaches. Uncovering the mechanisms underpinning initiation, progression, and promotion of tumour development are fundamental to the rational pursuit of targeted therapeutics. In particular, the role of inflammation in these mechanisms continues to be of interest.

**Figure 1:**
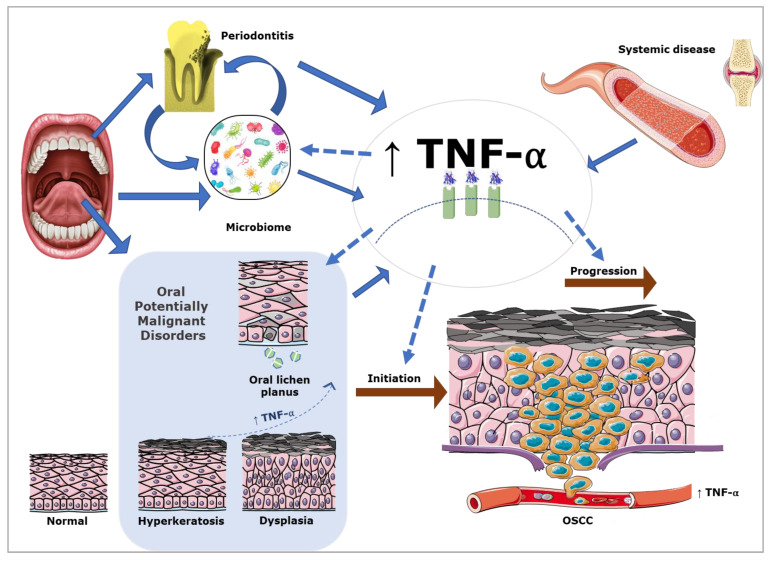
TNF-α has a role in the initiation and progression of OSCC. In the oral cavity, TNF-α expression is also influenced by the oral microbiome, the presence of other oral mucosal and periodontal disease, and systemic disease.

There is increasing evidence of an association between OSCC and chronic inflammation. Particularly relevant is an observed progressive increase in inflammatory infiltrate alongside increasing grades of epithelial dysplasia to OSCC [4]. The microenvironment of chronic oral inflammatory conditions such as oral lichen planus have been established to contain cytokine infiltrates, reactive oxygen species, and transcription factors capable of inducing proliferation, epithelial-to-mesenchymal transition, and invasion [4]. Microarray analysis in OSCC cell lines and OSCC patient samples have also identified dysregulation of the many genes involved in inflammation, wound healing, angiogenesis, and growth regulation [5]. Tumour associated-inflammatory cells and stromal cells are essential residents of the tumour microenvironment and have an influence on OSCC genesis, proliferation, survival, and ability to invade and metastasize [4]. Pro-inflammatory mediators, such as interleukin (IL)-6, IL-8, and tumour necrosis factor (TNF)-α, have been demonstrated to be elevated in oral cancer patients. Increased TNF-α in the tumour microenvironment has been demonstrated to favour invasion through promotion of pro-inflammatory, pro-invasive phenotypes of OSCC cells and paracrine mechanism mediated recruitment and activation of inflammatory cells [6,7]. The broad and multifaceted role that TNF-α plays in many inflammatory conditions presents an obvious confounder, particularly with demonstrated increased TNF-α levels in common oral disease states. This paper explores the current links between TNF-α and OSCC (Figure 1).

## 2. TNF-α, Cancer and SCC

TNF-α (also named TNF, cachexin, and cachectin) has been implicated as a large player in several inflammatory, infectious, metabolic, and neoplastic diseases. TNF-α plays a central role in the onset of immune response. It is predominantly secreted by macrophages but is also produced by other immune and non-immune cells, including fibroblasts, muscle, and endothelial cells [8,9]. It affects almost every cell type and is involved across several biological processes of cell growth, differentiation, metabolism, and death, as well as coagulation [10].

TNF-α is a trimeric cytokine and exists in two isoforms—a transmembrane form and a soluble form (mTNF and sTNF, respectively) [11,12]. The two known TNF receptors, TNF receptors 1 (TNFR1) and 2 (TNFR2), are differently activated by the TNF isoforms and have different ligands but have interconnected pathways and effects which can appear paradoxical. TNFR1 is found in all cell types and has a death domain with signalling proteins that link it to cytotoxic pathways as well as pathways that activate the nuclear factor of kappa B (NFkB) factors as well as MAP kinases [13]. Activation of TNFR1 can also stimulate downstream cell survival mechanisms through suppression of its cytotoxic signalling, and default TNFR1 activity is actually pro-inflammatory [9]. TNFR2 does not have a death domain but can stimulate NFkB signalling and various kinases. TNFR2 is mainly found in immune cells, where it modulates immune response and inflammation but can be induced on non-immune cells such as fibroblasts.

TNFα has been highlighted as a crucial mediator of cancer-related inflammation and thus its targeting may be a key to oncological therapy resistance. Expression and induction of TNFR1 and TNFR2 on immune stromal cells within the tumour microenvironment has been shown to promote tumour growth and progression. Further, their induction of immune suppressor cell differentiation also leads to evasion of tumour surveillance [14,15].

TNF-α has been linked to several human cancers, including breast [16], gastric [17], pancreatic [18], ovarian [19], endometrial [20], prostate [21], bladder [22], colorectal [23], oral [24], and liver [25]. It has also been detected in leukemias and lymphomas. Different pathways, e.g., p38 MAPK, Erk1/2, β-catenin, NF-κB, and GM-CSF, among others, have been found to be involved in different cancer types.

The discordant effects that TNFα can exert in cancer depend on a multitude of factors that include its concentration and the ratio of its two isoforms (mTNF, sTNF), among others. For example, one of the main characteristics of TNF superfamily members is their duality. Being able to act as both ligands and receptors creates a peculiar phenomenon known as “reverse signalling” [26]. TmTNFα can therefore signal outside-to-inside back to the tmTNFα expressing cell while acting as a receptor, and although not fully characterised, this mechanism has been shown to be involved in immune system regulation. Conversely, the active soluble form, sTNFα, can exert a powerful autocrine, paracrine, and endocrine effect. Moreover, the latter can be obtained via proteolytic cleavage of tmTNFα by an enzyme known as TNFα Converting Enzyme [27,28].

Other factors that can modify the role of TNFα in cancer include caspase activation, the variable expression of adaptor proteins, and proteins from the Bcl-2 family [29].

TNF-α can therefore act either as a pro-tumoral cytokine, favouring cell proliferation, cell migration, angiogenesis, and diseases progression in a number of cancer types, or as an anti-tumorigenic biomolecule. A summary of TNFα effects in several cancer types can be found in the work by Mercogliano et al. [15].

TNF-α plays a critical role in the pathobiology of cancer. It has also shown to be a key player in head and neck SCC (HNSCC), where it can stimulate the expression of programmed death-ligand 1 (PD-L1), a ligand for PD-1 that is primarily expressed on activated T/B cells, monocytes, and a small percentage of thymocytes [30]. PD-L1 is a member of the CD28 receptor family and although it is only weakly expressed in healthy tissues, it is both inducible and constitutively expressed in a wide variety of solid and blood cancers. It has been shown that its intracellular upregulation can promote tumour formation [31]. Additionally, tissues isolated from HNSCC showed the ability to synthesize PD-L1 through an aberrant PD-1 signalling pathway. This, in turn, can result in tumour immunosuppression [32], suggesting a potential role for TNF-α in this biological event.

In contrast, a clear role in oesophageal SCC for tumour necrosis factor alpha-induced protein 8 (TNFAIP8) has been demonstrated. TNFAIP8 is a suppressor of TNF-α-mediated apoptosis, and its expression is induced by NF-κB activation. A retrospective study by Sun et al. showed that TNFAIP8 overexpression correlated with lymphatic recurrence in ESCC patients [33]. TNF-α has further been demonstrated to have a crucial role in skin SCC. Skin TNF-α is produced as a proinflammatory cytokine in response to ultraviolet B radiation (UVB) to facilitate UVB-induced apoptosis and therefore contributes to remove the damaged cells and thus diminishing tumour genesis. In contrast, murine TNF-α-knockout animal models have demonstrated that TNF-α is necessary for the early stages of skin carcinogenesis and the development of SCC [34]. Therefore, TNF is involved in both pro- and antitumorigenic functions in cutaneous SCC with the distinct effect exerted mostly being context-dependent [35]. There is robust evidence that TNF-α is a crucial player in several human cancers, although with very discordant effects on different types of cancer.

## 3. The Role of TNF-α in the Aetiology of OSCC

Polymorphisms affecting gene expression of cytokines related to inflammation, such as IL-4, IL-6, IL-8, IL-10, and TNF-α, have been strongly associated with an increased risk for OSCC [36]. Although further work is required to understand the complex mechanisms underlying tumour microenvironment responses in relation to inflammatory cytokines, evidence suggests that proinflammatory, proangiogenic, and immunoregulatory activity is present in squamous cell carcinomas and become part of the local tumour milieu. Nuclear factor-kappa beta (NF-κB) is an early response gene that is elevated with the use of tobacco as well as chronic inflammatory conditions. Promoter regions for the proinflammatory and proangiogenic cytokines of IL-4, IL-6, IL-8, IL-10, and TNF-α have all been found to have the common nuclear transcription factor of NF-κB. Studies have demonstrated that there is an increased level of TNF-α and inflammatory cytokines in oral squamous cell carcinoma compared with premalignant lesions or undifferentiated oral leukoplakia [37,38,39]. It has been postulated that proinflammatory cytokines contribute to the development of oral squamous cell carcinoma through a positive cell cycle upregulation within the tumour microenvironment. Aberrant activation of NF-κB leads to propagation of a continuous vicious cycle of inflammatory cytokines, proangiogenic factors, and anti-apoptotic factor upregulation [39]. In vitro studies have shown that TNF-α activation of the NF-κB pathway leads to increased protein expression of IKKβ and P65, enhancing the ability of oral squamous cell carcinoma cells to invade through the epithelial basement membrane, and thus increase their ability to metastasize [24]. NF-κB interacts with important cell cycle proteins, such as cyclin D1, cyclin E, and c-myc, to promote tumour cell proliferation [40] and promotes survival and inhibits apoptosis through Bcl-2 and Bcl-xL, which regulates apoptosis [41]. NF-κB has been found to regulate the expression of matrix metalloproteinases, in particular MMP9, that enhances the invasion of tumour cells. Several studies have shown that expression of Snail, Slug, ZEB ½, and Twist 1 are altered through NF-κB activation [42,43,44]. Further, TNF-α upregulated Snail, Slug, ZEB ½, and Twist 1 are dependent on NF-κB activation and lead to cancer cell epithelial-mesenchymal transition, shown to be related to increased invasion and metastasis. However, these results were observed in a non-small cell lung cancer in vitro cell model requiring further extrapolation to oral squamous cell carcinoma [45].

## 4. The Role of TNF-α in the Promotion of OSCC

Regarding the ability of OSCC to invade and metastasize or be enhanced by the presence of inflammatory mediators [46,47], TNF-α is worthy of particular attention in OSCC, with its presence demonstrated to enhance cell proliferation and its downregulation demonstrated to inhibit proliferation and migration in other carcinomas, both in vitro [48] and in vivo animal models [49].

Although the mechanism of action through which TNF-α promotes these phenomena has not yet been elucidated, there is evidence to demonstrate that the NF-kB signalling pathway, activated by TNF-α, may be key to the promotion of invasion and migration [50]. In vitro studies conducted by Daofang Tang et al. [24] on OSCC cell lines demonstrated that stimulation with 10 ng/mL of TNF-α at several time points (3, 6, 12, 24, 48, and 72 h) produced a statistically significant increase (after the 3rd time-point) of the p65 protein and of the Ikβ kinase, which, by phosphorylating the inhibitory protein Ikβ, frees the NF-kB heterodimer from its inhibited state and allows it to carry out its pro-inflammatory action [24]. The same mechanism was confirmed by in vivo experiments on mice, demonstrating that selective inhibition of NF-κB suppresses bone invasion in a model of mandibular invasion by OSCC [51], more specifically, over a period of 3 weeks, using a model of mandibular invasion by squamous cell carcinoma cell line (SCCVII cells), injected three times a week with NF-κB essential modulator binding domain (NBD peptide), or control (a mutant NBD peptide) [52]. In all mice, an extension of bone invasion by OSCC was seen; However, interestingly, in the group of mice in which NBD peptide was injected, zygoma destruction was significantly suppressed [52]. This evidence gives strong evidence that stimulation of the NF-kB signalling pathway, induced by TNF-α, promotes the endosseous invasion of OSCC tumour cells.

A further mechanism of action through which TNF-α can be considered as a promoter of OSCC cell invasion and migration has been demonstrated in an in vitro study showing that TNF-α is able to activate the transcriptional repressor Snail that down-regulates E-cadherin and induces the mechanism of epithelial-connective tissue transition [53]. Analysing three human OSCC cell lines treated with or without TNF-α (10 ng/mL) for 72 h demonstrated, via RT-PCR and Western blot analyses, that Snail was strongly activated by TNF-α rapidly, within the first 30 min of treatment [53]. This study further showed, by Western blotting analyses, that the NF-κB pathway contributed to the activation of this mechanism [53].

The aforementioned mechanisms discussed clearly link the role of TNF-α with enhanced metastatic potential and highlight the ability of TNF-α to induce the malignant cell epithelial-connective tissue transition in OSCC. In contrast to the above, a recent study has demonstrated cross-talk between the macrophage-related inflammatory cytokines and the migration of cancer cells [52]. IL-6, IL-1β, and TNF-α effected the migration rate of both highly and poorly differentiated OSCC cell lines, CAL27 and SCC25, respectively, demonstrating that only IL-6 exerted a significant increase in cell migration rate, whereas TNF-α caused only a slight increase in migration rate and exclusively only in highly differentiated OSCC cell lines [52]. These results would indicate that, potentially, IL-6 has greater importance in migration and possibly metastases, and yet in those well-differentiated OSCC cells, previously thought to have low metastatic potential, perhaps the role of TNF-α is critical. Obviously, further unclarified bio-molecular mechanisms of signal transduction regulation related to TNF-α in the promotion of OSCC requires further exploration.

## 5. OSCC Prognosis and TNF-α

**Figure 2:**
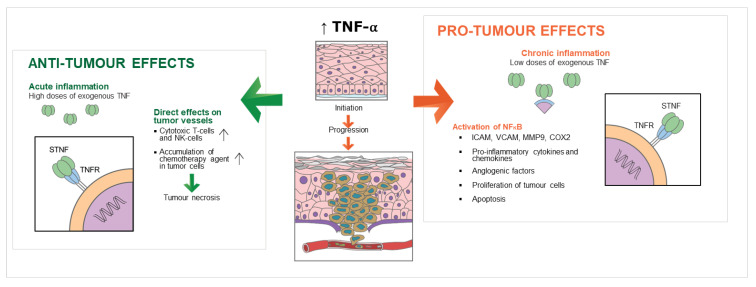
Anti-tumour and pro-tumour effects of TNF-α in OSCC make prognostic attribution challenging.

Prognosis is defined as the predicted course of a disease process and the anticipated outcome expected from treatment. In OSCC, TNM staging (tumour-node-metastasis), cancer staging, and certain conventional histological tumour grading have been utilized for therapeutic decision making but have been found to be imperfect predictors of prognosis. Several biomarkers have been discovered that appear to correlate with tumour aggressiveness and potentially prognosis of OSCC. These include epidermal growth factor, p53, cyclooxygenase-2 (COX-2), and matrix metalloproteinase-9 (MMP-9) [54,55,56,57]. TNF-α is another important biomarker being investigated in OSCC and head and neck cancers in general. Very little evidence exists regarding the potential role of TNF-α and OSCC prognosis. Its role as a pro-inflammatory cytokine makes it a possible promoter of cancer or a potential cancer suppressor due to its pro-apoptotic effects [58]. These complex functions and interactions make it problematic to attribute prognosis to TNF-α (Figure 2).

An indirect way of determining the importance of TNF-α in the prognosis of cancer is by measuring TNF-α converting enzyme (TACE) activity. TACE belongs to a large group of type I integral membrane proteins known as ADAMs (a disintegrin and metalloproteinase). These proteins are involved in a variety of cellular processes, including conversion of TNF-α as well as several other membrane-anchored proteins, including both epidermal cell growth factors and TNF family receptors, to their active form [59,60]. TACE is the enzyme responsible for proteolytic cleavage of the membrane bound precursor protein of TNF-α to release the biologically active form of TNF-α protein (17-kDa), a process known as sheddase. In human carcinogenesis, increased expression of TACE mRNA expression has not been demonstrated to correlate with cancer clinical stage or aggressiveness [61]. However, measuring the TACE active form and its sheddase activity using a modified TACE sheddase assay might be a better indicator of its role in tumourigenicity [60]. The study by Ge et al., 2009, measured TACE protein levels and TACE sheddase activity in head and neck squamous cell cancer cell lines (PCI-4A, PCI-4B, PCA-15A, PCI-13, and PCI-13), fresh head and neck cancer tissue samples (collected from 63 patients with samples from primary tumours, recurrent tumours, and lymph node metastases from a variety of head and neck sites), and compared them with fresh samples of normal oral keratinocytes. Using Western blot analysis, the authors demonstrated significantly higher expression of both immature and mature TACE protein levels in human head and neck squamous cell cancer cells, and fresh head and neck cancer tissue samples compared to normal oral keratinocytes. Additionally, TACE protein levels and TACE sheddase activity were both higher in T3/T4 tumours compared to T1/2 tumours; however, the difference was only statistically significant in TACE sheddase activity, which was also significantly higher in cases with lymph node metastasis and those that had recurrence after initial treatment [61]. These findings demonstrate that TACE sheddase activity levels in head and neck cancers are biologically and clinically relevant and may be a significant biomarker in discriminating between cancers more likely to recur after initial treatment and hence have a worse prognosis than those with a lower chance of recurrence and a better prognosis. Hence, the activation of TNF-α by TACE sheddase activity may be an important prognostic marker to consider as a reflector of disease aggressiveness for OSCC [61].

Identifying more specific genotypic expression of TNF-α from serum samples of head and neck cancer patients may also provide another avenue to guide prognosis. In a study by Santana et al., 2021, blood collected from 163 oral and oropharyngeal SCCs and 143 healthy controls was genotyped for PON1, TNF-α (two different genotypes), and TGF-β single nucleotide polymorphisms [62]. They found that the rs1800629 genotype of TNF-α was more frequently found in more advanced stage tumours, and those with poorer survival. In contrast, another genotype of TNF-α was more frequently found in the serum of patients with lip SCCs and clinical stage I and II tumours. Similar findings are being identified in other types of cancers. In patients with breast cancer, elevated serum levels of TNF-α were not necessarily found when comparing early ductal carcinoma patients with healthy controls. However, serum TNF-α levels were found to be significantly elevated in higher grade breast cancers and those with lymph node metastases compared with earlier stage I cancers [63]. Identifying elevated serum TNF-α levels may signal higher grade disease; however, this has not as yet been demonstrated in OSCC.

Recent studies have also demonstrated interesting findings when investigating the potential role of TNF-α and its family proteins to predict prognosis, tumour immune characteristics, and immunotherapy response in several cancer types. Although these studies have not been replicated in OSCC, they pose an interesting avenue for future research. The family proteins include the TNF receptor superfamilies that are composed of 19 ligands and 29 receptors. The communication between these pathways orchestrates inflammation and controls cell survival, proliferation, and differentiation. Gene expression, overall survival rate, and somatic mutation data were collected for 516 colorectal cancer cases from the National Cancer institute GDC data Portal (TCGA) to identify the TNF family genes that are risk factors for poor overall survival and the genes that are protective [64]. Out of 47 well defined TNF genes, nine genes were found to be relevant to overall survival, four as risk factors, and five were protective. A TNF family-based signature (TFS) was generated from these genes, and the patients were stratified into low and high risk. Low risk (low TFS score) patients had a better overall survival, and it is believed this is due to a high infiltration of resting CD4 memory T cells and resting dendritic cells, with few immune escape phenotypes, rending the tumour more sensitive to immunotherapy. A high TFS score was associated with high infiltration of regulatory T cells, non-activated macrophages, natural killer cells, and immune escape phenotypes. This led to poor response to immunotherapy and increased metastasis-related pathways [64]. A similar study evaluating the potential for a similar TNF signature for small-cell lung cancer and by stratifying patients into low and high risk based on their TFS score, both prognosis and response to chemotherapy was able to be predicted [65]. Within the same TCGA database, there is a similar comprehensive data series for over 2700 head and neck cancers. Performing a similar analysis of TNF family genes and development of a TFS score for OSCC may also demonstrate an important link between these genes and OSCC prognosis and potential response to immunotherapy.

## 6. TNF-α as a Biomarker in OSCC

Studies have considered TNF-α within biofluids (Table 1) and tissue as a potential biomarker for early detection of OSCC, staging, and differentiation. The broad and multifaceted role that TNF-α plays in inflammatory states presents an obvious confounder.

Whole saliva produced from the major and minor salivary glands are secreted fluid transported from serum as well as from surrounding glandular tissues. Saliva is composed of the secretions from salivary glands as well as oral mucosa, periodontium, and oral microflora that contribute to the final content of whole saliva [66]. The heterogenous nature of saliva, coupled with its ease of sampling, makes it an attractive prospect in the diagnosis of salivary gland disorders and oral diseases, particularly that of OSCC. A prospective cohort study investigating the use of saliva analysis involving multiple biomarkers including TNF-α for the early detection of OSCC in OSCC patients compared to controls showed that TNF-α as a saliva biomarker had a sensitivity of 39% and a specificity of 100%, with levels of TNF-α having a statistically significant difference in saliva compared to plasma of OSCC patients compared to controls [67]. In this study, samples were collected from three groups: control patients (n = 24), stage I/II OSCC (n = 22), and stage III/IV (n = 19), and multiple salivary markers were analysed. Regarding TNF-α, control patients had a TNF-α level of 8.6 pg/mL ± 7.27, whilst patients with OSCC had levels of 27.75 ± 30.94 pg/mL [67]. Altered salivary cytokine concentrations due to dental and oral inflammatory conditions remain a relevant confounder, and queries whether serum based biofluid sampling presents a more reflective sample remain, although serum has the drawback of not being as location specific as saliva. Multiple additional studies have found similar results demonstrating that levels of TNF-α and other cytokines, such as IL-6 and IL-8, are present in the saliva of patients with OSCC at significantly different concentrations compared to healthy controls [39,68,69,70,71].

Interestingly, cytokines in salivary levels increased regularly when compared with well differentiated to poorly differentiated oral squamous cell carcinoma. Levels of IL-6, IL-8, TNF-α, IL-1β, TNF-α, and IFN-γ were raised in early stage OSCC when compared to healthy controls [67]. Salivary and serum samples of patients analysed for TNF-α levels was able to differentiate healthy samples, premalignant lesions, and OSCC [71]. Levels increased in patients from premalignant lesions to OSCC, with a higher increase in stage 4 OSCC clinical staging. Levels of TNF-α were higher in salivary samples compared to serum samples, and this is concordant with other studies [67,68,72]. Multiple studies have also demonstrated the correlation of higher levels of TNF-α production in patients with OSCC compared to premalignant lesions and healthy controls, specifically in salivary samples with positive correlation of histological grading in OSCC [38,73,74]. Salivary TNF-α samples have been shown to have the potential for non-invasive monitoring of premalignant conditions during routine oral cancer screening [37,75]. However, further research is required in the field of salivary biomarkers as there is a wide variation in the average levels of salivary cytokines in oncologic patients and healthy controls [76].

**Table 1:** TNF-α levels as a liquid biopsy biomarker in OSCC and premalignant lesions and disease.

Outcome	Sample Type	Sensitivity/Specificity of Test	Levels Reported
Diagnosis and levels			
Normal	Saliva	Sensitivity 39%, Specificity 100% for diagnosis of OSCC [67]	4.5 ± 2.5 pg/mL [71] 38 ± 3.23 pg/mL [75] 3.0 ± 1.9 pg/mL [38] 4.1 ± 2.1 pg/mL [74] 8.6 ± 7.27 pg/mL [67]
	Blood (Serum or Plasma)	Sensitivity 39%, Specificity 100% for diagnosis of OSCC [67]	3.9 ± 2 pg/mL [71] 12.7 ± 4.89 pg/mL [77] 10.10 ± 6.08 pg/mL [67]
Premalignant lesion	Saliva	Not described	136.8 ± 59.6pg/mL [71] 30 ± 3.01 pg/mL [75] 10.5 ± 7.4 pg/mL [38]
	Blood (Serum or Plasma)	Not described	180.1 ± 52.4pg/mL [71]
Premalignant disease	Saliva	Sensitivity 97%, Specificity 83% for diagnosis of OSCC [71]	126.8 ± 59.2 pg/mL [71]
	Blood (Serum or Plasma)	Sensitivity 72%, Specificity 75% for diagnosis of OSCC [71]	166.5 ± 49.4 pg/mL [71]
OSCC	Saliva		311.9 ± 95.3 pg/mL [71] 34 ± 21.58 pg/mL [75] 28.9 ± 14.6 pg/mL [38] 35.2 ± 51.8 pg/mL [74] 27.75 ± 30.94 pg/mL [67]
	Blood (Serum or Plasma)		225.1 ± 99.9 pg/mL [71] 45.8 ± 37.01 pg/mL [77] 11.65 ± 7.32 pg/mL [67]

TNF-α serum levels for the correlation of OSCC for diagnosis remains inconsistent. Schiegnitz et al., 2017, carried out a prospective cohort study investigating serum biomarkers (IL-6, IL-8, sIL-2R, and TNF-α) in patients with OSCC, oral lichen planus (OLP), and healthy controls and found no significant differences in TNF-α serum levels between controls, OLP, and OSCC patients [78]. Similar results were reported in a prospective cohort study investigating the use of serum IL-6 and TNF-α as a serum biomarker in patients [79]. The prospective cohort study compared 36 newly diagnosed OSCC patients with 31 healthy blood donors. High serum levels were defined as >6 pg/mL for IL-6 and >28.6 pg/mL for TNF-α, and serum samples were analysed using ELISA testing. Interestingly, and different from the later studies, the majority of patients had clinical stage 3 or 4 OSCC, with this study reporting a positive correlation. Additional studies have shown that TNF-α in serum may be higher or lower in OSCC patients compared to controls [75,77,79]. The contrasting data within the literature highlights the complexity of TNF-α serum levels, approaches to measurement, and possible inflammatory confounders. TNF-a serum level alone has not yet emerged as a consistent biomarker for early detection of OSCC.

There are two studies investigating TNF-α levels in tissue samples and peripheral blood or saliva looking for correlation. Healthy matched controls were compared with clinically and histopathologically confirmed cases of SCC in a case control cohort study (n = 75 per group) [80]. Results from this study found that TNF-α transcript expression in OSCC patients was 2-fold higher compared to matched controls, as well as levels of IL-1b (1.91 fold), TFG-b (3.72 fold), and IL-10 (2.25 fold). Interestingly, peripheral blood levels of IL-10 and TGF-β were increased compared to matched controls, but other proinflammatory cytokines, including TNF-α, were comparable. These results reflect that the tumour can secrete proinflammatory cytokines, including TNF-α and IL-1b, contributing to the tumour milieu and propagation of the proinflammatory cascade. Samples of mucosa and saliva were taken from patients with a diagnosis of OSCC or oral potentially malignant disorders (OPMDs) in a cross-sectional cohort study sampling mucosa and saliva from patients (n = 60); saliva was compared with healthy matched volunteers [81]. Histological samples were analysed using immunohistochemistry, and saliva was analysed using ELISA. Immunoreactivity was significantly higher for TNF-α in th eepithelium and stroma of oral epithelial dysplasia (OED) compared with normal mucosa from peripheries of the tissue samples. Another cytokine that was significant was the presence of IL-8, which was higher in the stroma of OSCC compared with normal mucosa. Regarding the saliva, ELISA analysis levels of IL-6 (*p* = 0.0012), IL-8 (*p =* 0.0000), and TNF-α (*p* = 0.0492) were higher in patients with OSCC compared to healthy controls and patients with oral leukoplakia without associated dysplasia. The results of the above study are promising as they represent a positive correlation with IL-8 and TNF-α for tumour expression and salivary expression. Further studies are required to corroborate these results but constitute a step forward towards a non-invasive bedside/chairside adjunct to differentiate dysplasia or OSCC from suspicious oral lesions.

## 7. The Oral Microbiome and TNF-α in OSCC

There are over 770 bacterial species in the oral cavity [82], in addition to candidate phyla radiation (CPR) of bacterial organisms, bacteriophages, fungal genera, and viruses [83]. A more diverse bacterial microbiome, with higher numbers of operational taxonomic units (OTU) demonstrated by high throughput sequencing, was observed in healthy individuals compared to samples from individuals with OSCC [84], with similar findings of the fungal mycobiome [85]. Bacterial populations have also been shown to change dynamically with the progression of OSCC [86]. TNF-α primarily exerts its effects through the NF-kB pathway, which itself is significant in bacterial-associated inflammatory response and is upregulated in OSCC [87]. Both bacterial metabolite-sourced and TNF-α triggered reactive oxygen species (ROS) can promote DNA damage and angiogenesis, resulting in OSCC growth and progression [88].

The production of pro-inflammatory cytokines, such as interleukin IL-1b, IL-6, and TNF-α, is enhanced by common lipopolysaccharides and bacterial endotoxin of the pathogens associated with periodontal and endodontic disease (e.g., *P. gingivalis* and *F. nucleatum*) and are considered responsible for immune related periodontal tissue damage [83]. In vitro, OSCC cells infected with *P. gingivalis* showed an overexpression of TLR2, which in turn promoted the overexpression of MMP9, TNF-α, and IL-6 that augmented the proliferation and growth of infected cells. Further, *P. gingivalis* infection associated increased transcription of TNF-α coincided with fibroblastic phenotypic shifts, the expression of stem-cell like properties, and higher migratory invasive capacity of OSCC cells [89,90]. In vivo, chronic infection with *F. nucleatum* and *P. gingivalis* in a murine model of periodontitis-associated oral tumorigenesis demonstrated stimulation of OSCC induction and proliferation through direct interaction with oral epithelial cells via toll-like receptors [91].

TNF-α levels have been demonstrated to be increased in the gingival crevicular fluid and serum of individuals with chronic periodontal disease. Increased levels of TNF-α has also been highlighted as a link between periodontal disease and Diabetes Mellitus [92], as well as other inflammatory conditions such as rheumatoid arthritis [93]. Demonstration of the role of TNF-α in bone resorption by increasing osteoclastic activity and deceasing osteoblastic activity has shared relevance to both periodontal disease and OSCC [94]. An in vivo murine model of OSCC with some animals having periodontitis-associated bacteria addition, demonstrated increased alveolar bone resorption in the addition group as well as increased tumour mass and tumour growth rate that paralleled the significant upregulation of TNF-α [95].

Candida has been associated with the progression of OSCC in vitro and in vivo [96], and a significant association between oral cancer occurrence and Candida oral colonization in humans has been demonstrated [97]. Endothelial cells are stimulated to synthesize TNF-α in response to in vitro infection with *C. albicans* [98]. Interestingly, TNF-α has been demonstrated to suppress the hyphal formation of *C. albicans* blastospores directly and dose-dependently. In vivo, the oral administration of TNF-α significantly reduced the *C. albicans* CFU and the in-situ number and size of *C. albicans* observed histopathologically on the tongues of treated mice [99]. Interaction with, and contribution from, the oral microbiome mediated by TNF-α continues to be of relevance in the mechanisms of OSCC initiation, development, and progression.

## 8. TNF-α in Oral Potentially Malignant Disorders

Interest in the utility of TNF-α as a biomarker for early cancer detection connects with its discovered role in stepwise inflammation-mediated carcinogenesis [39]. Oral potentially malignant disorders (OPMDs) are a group of mucosal conditions characterized by an associated risk of progression to OSCC. These conditions include, but are not limited to, leukoplakia, erythroplakia, lichen planus (OLP), and oral submucous fibrosis. These conditions are described as variably featuring chronic inflammation [100,101]. The TNF-α encoding gene (−308 G/A) may be associated with the development of OPMDs with polymorphisms significantly associated with the OPMD specimens [102].

The mechanisms of malignant transformation through its different stages, from non-dysplastic, hyperkeratosis, and oral dysplasia to oral squamous cell carcinoma (OSCC) are still unknown. However, it is postulated that chronic inflammation, consisting of various types of proinflammatory mediators, such as activated cytokines and chemokines, likely plays a role in this process [4].

TNF-α can influence the different stages of etiopathogenesis of OLP, divided into specific and non-specific mechanisms. However, the specific mechanisms involved in OLP are poorly understood, and it is proposed that an altered or a foreign antigen in the basal cell layer is a trigger to provoke a T cell-mediated autoimmune response against these cells. Some genes of heat shock protein (HSP) have been found in the TNF-α gene region that increase the expression of this protein on the keratinocyte surface and it heralds the role of TNF-α in OLP [103]; oral keratinocytes release multiples of pro-inflammatory cytokines such as TNF-α that contribute at different stages of OLP pathogenesis. Increased level of TNF-α in OLP activates the Nuclear Factor Kappa B (NF-Kb), which is a transcription factor, and this activation leads to provoke an abundance of the inflammatory cytokines and chemokines that eventually recruit immune cells in this condition [104]; nonspecific mechanisms are also a part of the pathogenesis of OLP. These mechanisms seem to have been started by the degranulation of mast cells and the release of proinflammatory mediators. Mast cells release TNF-α that increases the production of matrix metallopeptidase (MMP), which leads to the destruction of the basement membrane [104]. In addition, the increasing vascular permeability and proliferation of endothelial cells, mediated by TNFα in the OLP, can be an indicator of the role of this proinflammatory cytokine in the non-specific mechanisms involved in this condition [103].

A meta-analysis demonstrated that TNF-α levels in OLP were significantly increased in various tissue types, suggesting that this increased level may be effective in initiating the disease and activating auto-inflammatory mechanisms [104]. In particular, polymorphism in the TNF-α gene was one of the factors [104]. Clinically, the elevated salivary TNF-α levels observed in patients with OLP is proposed to herald the onset and progression of the disease, and suggest potential utility in diagnosis and prognosis [105].

Cytokines may be involved in anti-tumour mechanisms or enhance the malignant transformation and tumour growth. Immunoreactivity using IHC analysis has shown significantly higher expression of TNF-α in OSCCs and OPMDs with and without dysplasia compared to normal controls. Interestingly, comparing leukoplakias with dysplasia to OSCC, significantly less TNF-α expression in the stroma (*p* = 0.0102) was found, which may support its role in progressive pre-malignancy [81].

Immunoregulatory events that occur downstream of TNFR1 may be critical for the development of OSCC in OPMDs. Increased TNF-α and TNFR1 expression, along with increased recruitment of CD45+ inflammatory cells, was observed in samples from OPMDs, which went on to become OSCC when compared to non-progressing OPMD samples [100].

Induced OSCCs in a 4-NQO mouse model of oral carcinogenesis also demonstrated upregulation of TNF-α and TNFR1 expression. Further, neutralization of TNF-α lead to in vivo decreased serum cytokines, inhibited development of invasion, and reduced neutrophils in the tumour microenvironment [100].

Evidence has suggested that the NF-κB–dependent cytokine levels (TNF-α, and additionally IL-4, IL-6, IL-8, and IL-10) are elevated in both the saliva as well as the tissue specimens from patients with oral premalignant lesions [101] (Table 2). Salivary TNF-α was significantly higher in patients with OSCC compared to patients with OPMDs without dysplasia or OLP with a trend of an increase in dysplasia, which was not significantly different from OSCC [81]. Further, salivary levels of TNF-α have been demonstrated to be markedly higher in moderate to severe levels of dysplasia compared to mild ones [101]. Others have found no significant difference between levels of dysplasia (*p* = 0.08) and significantly elevated salivary TNF-α levels in patients with OSCC compared to normal tissue, as well as oral dysplasia with or without dysplasia (ANOVA *p* < 0.001) [39]. These conflicting results make it difficult to conclude if TNF-α levels are useful for monitoring the malignant transformation of oral leukoplakia.

Biologic agents targeting TNF-α are currently in clinical use for immune-mediated inflammatory rheumatological and gastrointestinal disease. Current FDA approved drugs are monoclonal antibodies (infliximab, adalimumab, golimumab, and certolizumab) or receptor fusion proteins (etanercept). There is accumulating evidence that TNF-α may be a target for solid tumour therapy, with clinical trials of etanercept showing disease stabilization or partial improvement in patients with metastatic breast cancer [106]. There are only limited reports of the use of TNF-α antagonists in OSCC (Table 2).

Small animal studies such as these are encouraging, but many therapeutic targets have fallen short of the clinical bedside and remained on the lab bench or fallen victim to the translational canyon. As such, further studies in larger animal models/clinical trials are required to establish if TNF-α antagonists have a role to play in the management and prevention of OSCC clinically.

## 9. Conclusions

TNF-α has proven to be an important cytokine in the inflammatory cascade and may yet be a potential target in OSCC detection, prevention, and treatment. TNF-α has been observed in a participant role in OSCC but additionally in pre-malignant mucosal and microbiome-related oral disease, complicating its allocation to a biomarker role. Despite some promising results, further work is required in larger clinical populations to correlate TNF-α with prognosis and suitable utility as a biomarker, and ultimately if the TNF-α antagonism can play a novel adjunctive therapeutic role in the prevention or treatment of OSCC.

## Figures and Tables

**Table 2:** TNF-α inhibitor therapy in OSCC.

TNF-α Inhibitor	Mechanism of Action and Clinical Translation
ADALIMUMAB	Monoclonal antibody with TNF-α as a target. No clinical studies at present.
ETANERCEPT	Fusion protein produced by recombinant DNA. Reduces the efficacy of TNF and works as a TNF antagonist. No clinical studies at present.
GOLIMUMAB	Monoclonal antibody with TNF-α as a target. The TNF-α antagonist golimumab has been assessed in an experimental metastatic murine model in vivo, specifically using OSCC cells depleted of interferon induced protein with tetratricopeptide repeats 2 (IFIT2), a protein known to promote cell death via apoptosis. TNF-α antagonists reduced angiogenesis, tumour growth, and metastasis [106].
CERTOLIZUMAB	Pegylated monoclonal antibody directed against TNF-α. No clinical studies at present.
INFLIXIMAB	Chrimeric monoclonal antibody directed against TNF-α. No clinical studies at present.
